# Small-Angle Neutron Scattering Insights into 2-Ethylhexyl Laurate: A Remarkable Bioester

**DOI:** 10.1021/acssuschemeng.3c04736

**Published:** 2024-01-22

**Authors:** Oliver S. Hammond, Daniel C. Morris, Guillaume Bousrez, Sichao Li, Liliana de Campo, Carl Recsei, Michael Moir, Sergei Glavatskih, Mark W. Rutland, Anja-Verena Mudring

**Affiliations:** † Department of Biological and Chemical Engineering and iNANO, Aarhus University, Aarhus C 8000, Denmark; ‡ Department of Materials and Environmental Chemistry, Stockholm University, Stockholm 114 18, Sweden; § School of Chemical Engineering, University of New South Wales, Sydney 2052, Australia; ∥ Division of Surface and Corrosion Science, School of Engineering Sciences in Chemistry, Biotechnology and Health, KTH Royal Institute of Technology, Stockholm 100 44, Sweden; ⊥ Australian Centre for Neutron Scattering, ANSTO, Lucas Heights, New South Wales 2234, Australia; # National Deuteration Facility, ANSTO, Lucas Heights, New South Wales 2234, Australia; ∇ Department of Engineering Design, KTH Royal Institute of Technology, Stockholm 100 44, Sweden; ○ Department of Electromechanical, Systems and Metal Engineering, Ghent University, Ghent 9052, Belgium; ◆ School of Chemistry, University of New South Wales, Sydney 2052, Australia; ¶ Laboratoire de Tribologie et Dynamique des Systèmes, École Central de Lyon, Lyon 69130, France

**Keywords:** solvents, esters, lubrication, small-angle scattering, biobased materials, biodegradable

## Abstract

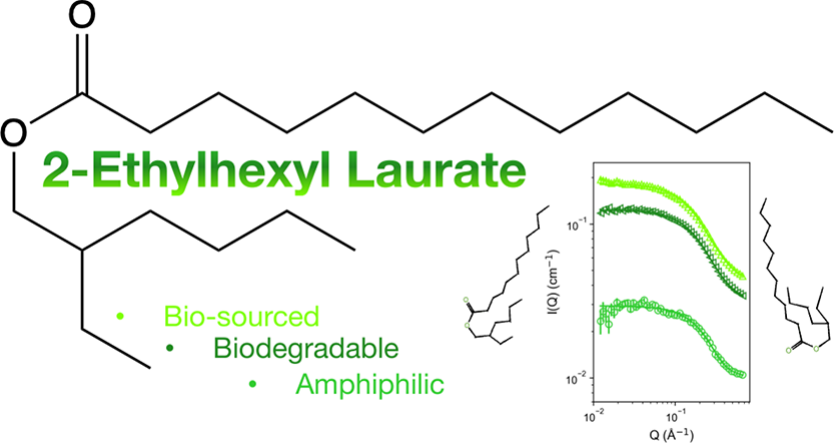

Commercial (protiated) samples of the “green” and biodegradable bioester 2-ethylhexyl laurate (2-EHL) were mixed with D-2-EHL synthesized by hydrothermal deuteration, with the mixtures demonstrating bulk structuring in small-angle neutron scattering measurements. Analysis in a polymer scattering framework yielded a radius of gyration (*R*_g_) of 6.5 Å and a Kuhn length (alternatively described as the persistence length or average segment length) of 11.2 Å. Samples of 2-EHL dispersed in acetonitrile formed self-assembled structures exceeding the molecular dimensions of the 2-EHL, with a mean aggregation number (*N*_agg_) of 3.5 ± 0.2 molecules across the tested concentrations. We therefore present structural evidence that this ester can function as a nonionic (co)surfactant. The available surfactant-like conformations appear to enable performance beyond the low calculated hydrophilic–lipophilic balance value of 2.9. Overall, our data offer an explanation for 2-EHL’s interfacial adsorption properties via self-assembly, resulting in strong emolliency and lubricity for this sustainable ester-based bio-oil.

## Abstract 2

Small-angle neutron scattering measurements of the sustainable biobased ester 2-ethylhexyl laurate (2-EHL) reveal surfactant-like configurations in the bulk, enabling self-assembly phenomena when deployed as a solvent or formulation ingredient.

## Introduction

There are few classes of chemicals with a more far-reaching impact on the global economy than liquid solvents, which contribute to the formulation and chemical synthesis of products essential to modern society, including mechanical lubricants, pharmaceuticals, and cosmetics.^1^ In a plurality of applications, a degree of solvent hydrophobicity is required to ensure that the active materials in the product are fully soluble or actively occupying stable separated phases in the case of (micro)emulsions;^2^ thus, these solvents must generally be neutral and nonreactive and contain predominantly nonpolar moieties such as long alkyl tails. Unfortunately, many oils possess undesirable traits, such as being toxic or nonrenewable, not aligning with the goals for sustainable development described by the UN in 2015.^3^ In this context, fundamental studies into sustainable solvents are enabling the targeted and informed deployment of sustainable, nontoxic, naturally sourced chemical compounds. While much of the “green solvent” limelight is often shone upon ionic liquids (ILs),^4^ there is significant space for simple biosourced materials to replace commonly used nonrenewable materials, such as petrolatum, paraffins, and mineral oils.

**Figure 1:**
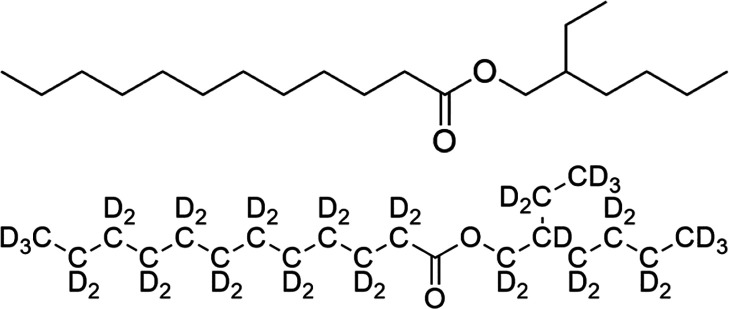
Chemical structure of fully protiated 2-ethylhexyl laurate (2-EHL) (top) and fully deuterated 2-EHL-*d*_40_ (bottom) solvents synthesized and studied here.

One such sustainable compound of interest is 2-ethylhexyl laurate (2-EHL), the ester of 2-ethylhexyl alcohol and lauric acid (Figure 1). 2-EHL is simultaneously neither mutagenic, genotoxic, irritating, nor sensitizing, and it has low toxicity: the dermal LD_50_ of ethylhexyl laurate in rats is >3 g kg^–1^ (by body weight).^5^ 2-EHL is composed of commodity chemical building blocks: 2-ethylhexanol, which naturally occurs in its (*R*)-form in plants and fruits and is produced at rates of >2 Mt per year, while high concentrations of lauric acid can be found in various species of commercially farmed products such as coconut, date, and oil palms, as well as in byproducts of the dairy farming industry,^6^ also amounting to megaton scales annually.^7^ Biodegradation of 2-EHL is rapid and reaches 91.7% within 28 days.^8^ 2-EHL can thus be legitimately considered as a sustainable “green bio-oil”.

In practice, preparations exist such as the enzymatic coproduction of 2-EHL alongside (*R*)-2-ethylhexanol, via the lipase-catalyzed acylation of racemic alcohol and vinyl laurate.^9^ Simple Zn-based homogeneous catalytic routes are also viable for this family of oils.^10^ Accordingly, 2-EHL and its sister esters are named in thousands of patents, which describe utilization in applications as broad as the formulation of eye makeup and skincare products such as cosmetic creams due to its skin-conditioning (emollient) properties, as well as in the paper industry, as an activity enhancer for pesticides, and in friction-reducing lubricant preparations.^9,11,12^ Combining its low viscosity, high penetration, and spreading effect, it is interesting for washing and cleaning products, polymers, adhesives and sealants, textile treatment products and dyes, lubricants and greases, plant protection products, pH regulators, water treatment products, and cosmetic formulations as a skin conditioner, moisturizer, and emollient.

Although its ester linkage renders 2-EHL a slightly more chemically active and polar material than conventional petrochemical alkane oils, there is good precedent for the usage of ester oils in lubrication.^13^ An advanced example can be seen in the use of 2-EHL as a green base oil, for the formulation of highly effective wear-reducing lubricants using solubilized ILs.^14^ Halide-free orthoborate ILs have been formulated for this purpose, and they degrade into friction-reducing, nonsacrificial surface tribofilms in concert with the 2-EHL base oil. Moreover, the lubricity of the adsorbed layer can be controlled by the application of an electric field.^15^

Despite (or perhaps due to) its widespread commercial intrigue, relatively little data beyond basic physical properties and safety information exist in the literature for 2-EHL.^5^ In the context of its structure (see Figure 1), this gap in the knowledge base is an important one to address due to two main factors: (1) 2-EHL contains a central ester linkage and therefore lies somewhere between a traditional linear alkane oil and a surfactant in terms of polarity and polarity distribution (i.e., the hydrophilic–lipophilic balance and dielectric constant). (2) 2-EHL contains a 2-ethylhexyl subunit, where the branching is known to alter properties,^16^ most famously seen in the drastic differences in self-assembled structures formed by the archetype dichain surfactant 2-ethylhexyl sulfosuccinate (AOT) and its derivatives.^17^ There are therefore several open questions regarding the bulk nanostructure and preferred configuration of 2-EHL molecules under standard conditions, which impact its properties and subsequent application as an ingredient. To clarify these questions, in this work, we will describe our measurements of pure 2-EHL using small-angle neutron scattering (SANS), applying H/D (^1^H/^2^H) isotopic substitution, to reveal the existence of any submicrometer bulk structure.

## Results and Discussion

**Figure 2:**
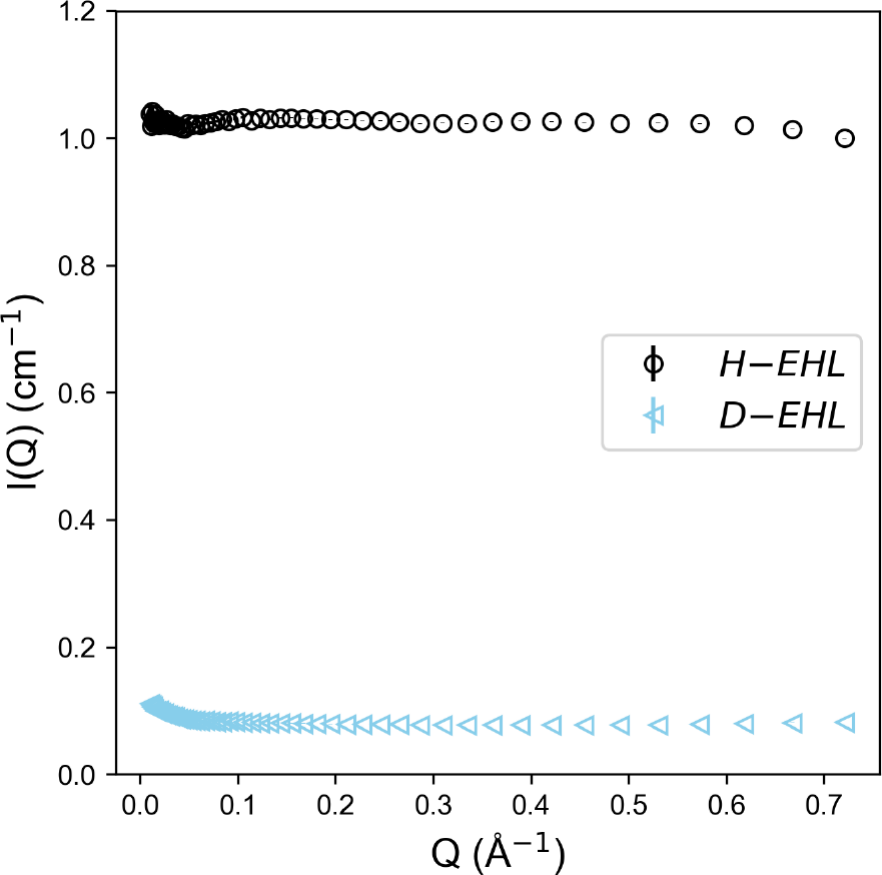
SANS data for pure H-2-EHL (black markers) and D-2-EHL (blue markers), showing a flat background signal for both.

In the first instance, pure examples of both neat H-2-EHL and D-2-EHL were measured using SANS (small-angle neutron scattering) on the BILBY SANS instrument (ANSTO, Australia);^18,19^ these data are shown in Figure 2. As expected, the constant incoherent background level *c*_0_ is substantially higher for H-2-EHL than for D-2-EHL, but it is challenging to compare this to theoretical predictions, i.e., from Cotton’s method, due to a lack of available isothermal compressibility (χ_*T*_) data in the literature for 2-EHL, in addition to poorly defined multiple scattering and thermalization contributions. Nevertheless, the *c*_0_ variation clearly demonstrates the high inelastic scattering contribution from H;^20^ samples of D-2-EHL prepared by hydrothermal deuteration (see the Supporting Information) were confirmed by mass spectrometry to contain 97.4 ± 2% D. These data sets are functionally flat, meaning that the scattering signal has no *Q* (scattering vector) dependence outside of the expected experimental error and noise. There is no structure evident under the given measurement conditions because there is no contrast between the scatterer and the bulk average; this would therefore be the same in the case of measurement by small-angle X-ray scattering (SAXS) or dynamic light scattering (DLS). Thus, contrast-variation SANS is the only current experimental method that can determine Å-to-nanometer scale structures for systems like this due to the squared contribution of the scattering length density (ρ) difference to the intensity, i.e., *I*(*Q*) ∝ (ρ_1_ – ρ_2_)^2^, considering the major difference between the scattering lengths *b* of H (*b* = −3.74 fm) and D (*b* = 6.67 fm).

**Figure 3:**
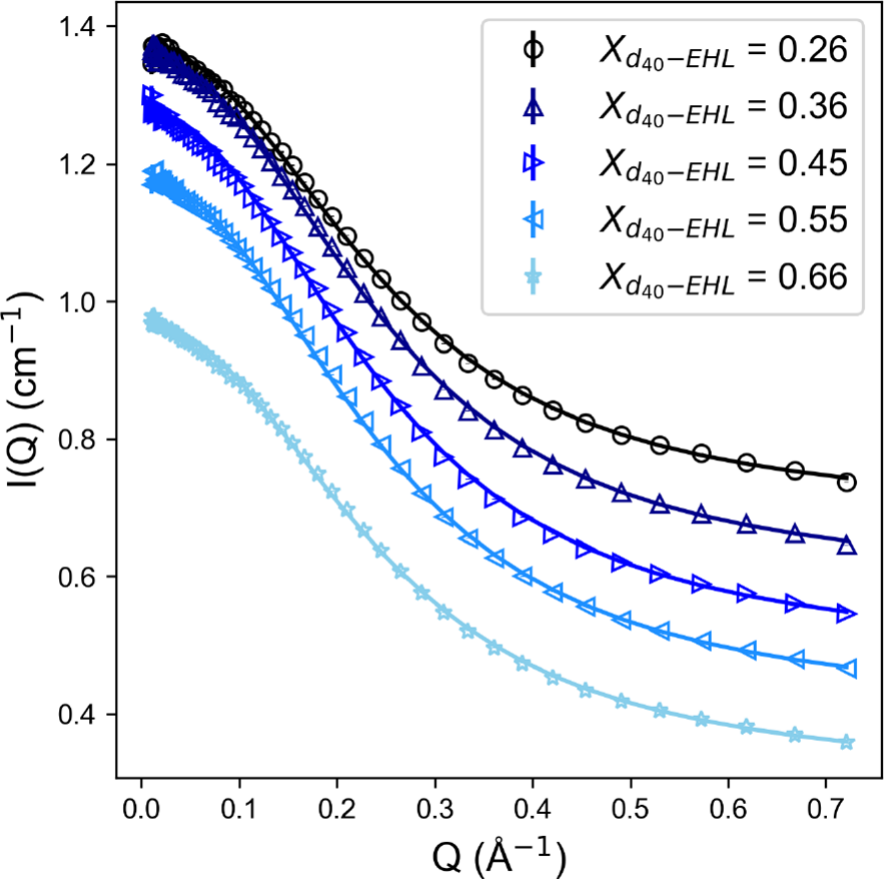
SANS data (markers) for isotopic mixtures of H-2-EHL and D-2-EHL across various mole fractions. Fits to the data using the Debye model are shown as lines of the same color.

A series of isotopic mixtures of H-2-EHL and D-2-EHL were thus measured using constant-wavelength SANS, shown in Figure 3; see the Supporting Information for a log–log plot of the same data and fits. Unlike the pure oils, there is a clear *Q* dependence of the measured SANS signal. Partial deuteration creates a large contrast differential between the bulk average and any structural disturbances. The data shown in Figure 3 therefore contain information on the local compositional fluctuations, which have been shown to be the dominant contributor to the scattering signal for alkane mixtures (exceeding the contributions from incoherence and local density fluctuations).^21^ Debye’s expression (eq 1) was therefore used to fit the 2-EHL scattering data as flexible Gaussian polymer chains, with the corresponding fitted parameters shown in Table 1.^22^ This methodology has been successfully applied before by Arleth and Pedersen to decane and isooctane systems^21^ and subsequently by Smith and Prevost to C_14_H_30_/C_14_D_30_ mixtures.^23^ The simple Debye model, with relatively few free fitting parameters, appears to represent a better fit to such systems than the random phase approximation (RPA). It is worth noting here that small-angle scattering data such as these are typically information-poor, and care must be taken so that data are not overinterpreted.^24^ Approaches from data science, such as Shannon’s theorem^25^ and Bayesian analysis,^26^ have been applied to analysis of small-angle scattering data, where they can be used to deduce parameters such as the number of Shannon channels (*N*_S_), describing the information content of a data set.^27^ These were calculated and are shown in Table 1 alongside the “number of good parameters” (*N*_G_), which verify that as the H content of samples is reduced, the data generally become more information-rich due to the lower *c*_0_, or in other words, the signal-to-noise ratio is improved in measurements of D-rich samples.
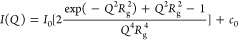
1

**Table 1:** Parameters Fitted to the H/D-2-EHL Mixtures Measured Here Using the Debye Model (Equation 1): The Radius of Gyration (*R*_g_), the Scale Factor for Coherent Scattering (*I*_0_), and the Constant Incoherent Scattering Background Contribution (*c*_0_)^a^

*X*_D-EHL_	*R*_g_ (Å)	*I*_0_ (cm^–1^)	*c*_0_ (cm^–1^)	*N*_S_	*N*_G_
0.26	6.446 ± 0.037	0.691 ± 0.002	0.682 ± 0.001	4.67	2.92
0.36	6.457 ± 0.034	0.779 ± 0.002	0.583 ± 0.001	4.75	2.83
0.45	6.486 ± 0.034	0.800 ± 0.003	0.479 ± 0.001	8.07	7.09
0.55	6.516 ± 0.033	0.777 ± 0.002	0.401 ± 0.001	7.92	7.07
0.66	6.527 ± 0.031	0.668 ± 0.002	0.302 ± 0.001	8.03	7.26

a The information content of the SANS data was calculated using BayesApp 1.1 and is shown as the number of Shannon channels (*N*_S_) and “number of good parameters” (*N*_G_).^28^

The average calculated *R*_g_ for the 2-EHL from these measurements was 6.49 ± 0.03 Å. To provide context for the fitted *R*_g_ values, the contour length (*L*_C_), describing the extended linear end-to-end distance of 2-EHL, was calculated to be 22.5 Å (comparable to a C_18_ chain of 22.6 Å), using bond length and angle parameters found in various common molecular dynamics force fields, such as OPLS and ILFF.^29−32^ Both the calculated *L*_c_ and fitted *R*_g_ perfectly match the values expected for the octadecane-like (C_18_H_38_) molecular size of 2-EHL by extrapolation from previous calculations and measurements of smaller linear alkanes^21,23^ while being substantially smaller than the “infinitely long hydrocarbon” example of polyethylene (PE).^33^ The fitted value of *c*_0_ decreases linearly with the mole fraction of D-2-EHL, signifying the expected reduction in incoherence as H is removed from the mixture.

**Figure 4:**
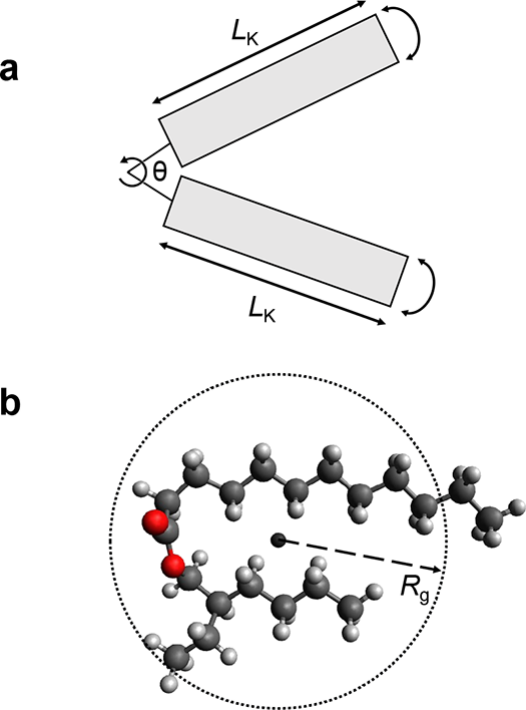
(a) Schematic representation of 2-EHL considered as two independently mobile, linear Kuhn segments of (persistence or average segment length) *L*_K_, shown in a surfactant-like configuration; (b) representation of 2-EHL in the proposed “surfactant-like” conformation, with the two long alkyl tails oriented linearly away from the more polar head group following energy minimization. The center of mass for this configuration was calculated using Avogadro software,^38,39^ with the radius of gyration (*R*_g_) from SANS (*ca*. 6.5 Å) drawn around this point.

Using eq 2, the average fitted radius of gyration (*R*_g_) determined from the SANS fits was then used to calculate the Kuhn length (*L*_K_) of 2-EHL:
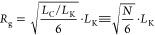
2where *L*_C_ is the contour length, *L*_K_ is the Kuhn length, and *N* is the number of Kuhn segments.^34^ From this expression, it is calculable that for 2-EHL, *N* = 2 and *L*_K_ = 11.22 Å. In other words, 2-EHL can be treated using the same framework as a compact molecular version of linear Gaussian polymer chains, with 2 “Kuhn-type” subunits, which are freely able to rotate and gyrate with respect to the other, without any energetic penalty. Surprisingly, this is only slightly smaller than *L*_K_ for some classic polymers, such as polydimethylsiloxane (PDMS)^35^ or polyethylene (PE),^36^ where *L*_K_ = 15.6 and 15.4 Å, respectively, but is larger than that calculated for dodecane (8.9 Å).^23^Figure 4a shows the Kuhn segment interpretation of 2-EHL structuring. In this framework, the structuring of the liquid phase can be considered as analogous to that observed for simple (i.e., PE/PDMS) polymer melts^35,36^ but with significantly smaller molecular weights and thus lower *R*_g_ values.

It is now possible to offer interpretations for the favored conformations of 2-EHL, based on the information gathered by Kuhn segment analysis of the SANS data. The experimentally measured *R*_g_ of 6.5 Å represents  of the fully extended molecular contour length, and this substantial difference must represent a high degree of molecular flexibility. If the two segments are statistically distributed, with θ ≤ 45° defining a “surfactant-like” 2-EHL monomer as shown in Figure 4a, then these configurations would comprise *ca*. 15% of the available orientational space. Simplifying this to a single configuration shown in Figure 4b and by calculation of its center of mass, a nearly perfect match is obtained for the experimental *R*_g_, without considering any further chain flexibility or conformations. We note that there may be several higher-energy accessible conformations, where the branched chain in particular is oriented differently. It is also important to acknowledge that these presented results are in the context of the commercially relevant racemic mixture of synthetic 2-EHL studied herein. Scalemic mixtures containing unequal quantities of (*R*) and (*S*) 2-EHL, or naturally sourced enantiopure samples, may yield different structural configurations and, therefore, different physical properties. Nevertheless, this proposed “surfactant-like” configuration of 2-EHL sees the polar ester linkage partially segregated from the apolar alkyl tails, which orient parallel and away from the oxygen-rich “head group”. This amphiphilic configuration being favored will confer (weak) surface activity (surfactant behavior), and thus, interfacial adsorption and self-assembly phenomena. This orientation has been previously proposed to explain the beneficial lubricative properties of 2-EHL when it adsorbs at metal interfaces, from experimental neutron reflectivity data investigating 2-EHL as a carrier oil for lubricant formulations.^13−15,37^ The potential to orient into surface-active molecular configurations, where self-assembly processes can actively occur, offers an explanation for the superior performance of 2-ethylhexyl esters in applications such as base oils for drilling fluids^12^ and skin moisturizing components in cosmetic formulations, when compared to traditional linear alkanes.^11^

**Figure 5:**
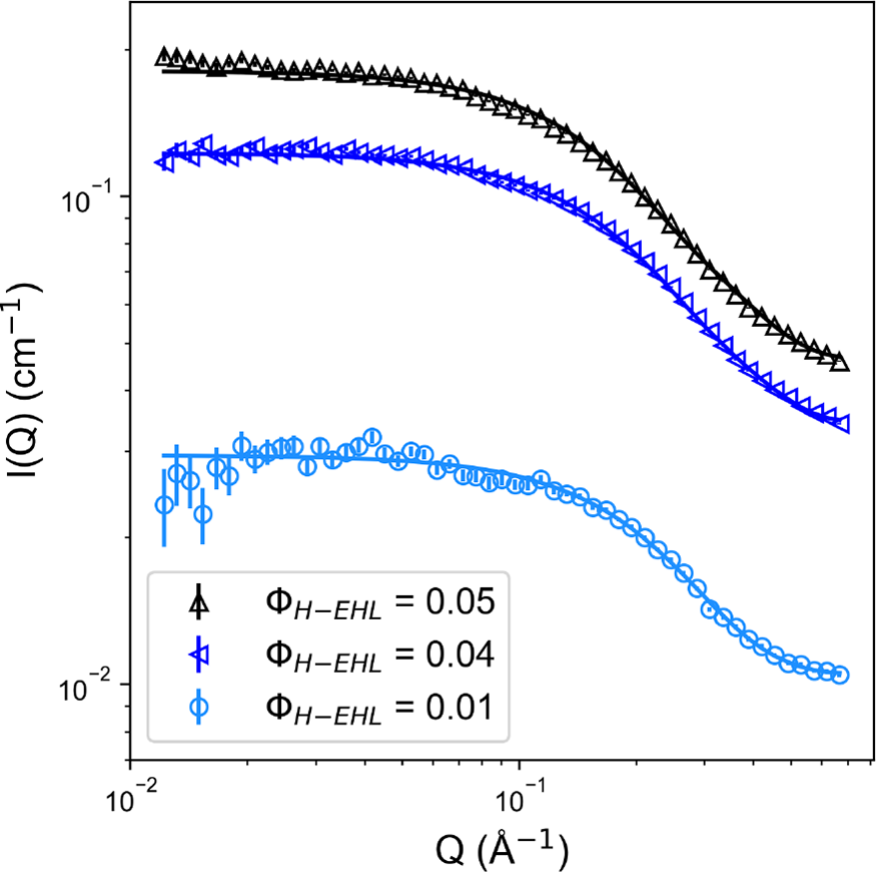
Small-angle neutron scattering (SANS) data for various volume fractions of H-2-EHL dispersed in *d*_3_-acetonitrile (markers) are shown alongside fits (solid lines) to a cylindrical model.

To further confirm the hypothesis that 2-EHL adopts surfactant-like configurations, blends were prepared of H-2-EHL with *d*_3_-acetonitrile as a model nonaqueous dispersant, up to the approximate room-temperature solubility limit (*ca*. 5 wt %). Further SANS measurements were taken of the mixtures (Figure 5), clearly showing structuring above the background. Samples fitted well to a cylindrical form factor (see the Supporting Information for further fitting details), with the dimensions of the self-assembled structures exceeding the individual molecular dimensions. Neglecting the slight concentration effect observed, this data set shows that, in acetonitrile, 2-EHL assembles into cylinders of 5.5 ± 0.4 Å mean radius and 22.8 ± 3.7 Å length, giving an average volume of 2125 ± 106 Å^3^ and thus a mean aggregation number (*N*_agg_) of 3.5 ± 0.2 molecules. The method of Glatter et al. was also applied to calculate *N*_agg_ directly from the background-subtracted forward intensity (*I*_0_),^40^ which yielded a comparable mean *N*_agg_ of 2.2 ± 0.3 molecules. Therefore, the self-assembly dimensions observed in acetonitrile show that the amphiphilic character of 2-EHL is sufficient for it to act as a small nonionic surfactant, as inferred from measurements of H-2-EHL/D-2-EHL mixtures.

## Conclusions

2-Ethylhexyl laurate (2-EHL) is an important and broadly applicable ester, which can be simultaneously biosourced and enzymatically synthesized and subsequently undergoes biodegradation in the natural environment. By measuring H/D mixtures of synthetic racemic 2-EHL using small-angle neutron scattering and analyzing this with classical polymer scattering frameworks, we have shown evidence for the “surfactant-like” or partially self-assembled molecular configuration of this vital ester. This configuration is implicated by Kuhn segment analysis, the measured *R*_g_ values, and further SANS measurements, which showed cylindrical 2-EHL aggregates forming in deuterated acetonitrile as a model dispersant. This intermediate nature, where 2-EHL can act as a nonionic surfactant with character of both a solvent and an amphiphile, is likely the explanation for the unique interfacial and lubrication properties of this molecule, which do not necessarily match the behavior expected from the low calculated hydrophilic–lipophilic balance (HLB) for 2-EHL of 2.9. The insights revealed here are thus of significant interest for the targeted formulation of ester-based sustainable lubricants and cosmetics.

## Supporting Information Available

Experimental (SANS and deuteration) details and characterization (PDF)
