# Supplementary material for: Small-Angle Neutron Scattering Insights into 2-Ethylhexyl Laurate: A Remarkable Bioester
Source: ACS Sustain Chem Eng. 2024 Jan 22;12(5):1816–21. doi: 10.1021/acssuschemeng.3c04736 (PMC10848283; doi:10.1021/acssuschemeng.3c04736)
Supplement: Supplementary file 1 — sc3c04736_si_001.pdf [file sc3c04736_si_001.pdf]

Supporting Information for:

Small-Angle Neutron Scattering Insights into 2-Ethylhexyl Laurate: A Remarkable Bio-Ester

Oliver S. Hammond[a,b] Daniel C. Morris,[c] Guillaume Bousrez,[a,b] Sichao Li,[d] Liliana de Campo,[e] Carl Recsei,[f] Michael Moir,[f] Sergei Glavatskih,[g,h,i] Mark W. Rutland,[*d,i,j] Anja-Verena Mudring[*a,b]

[a]Department of Biological and Chemical Engineering and iNANO, Aarhus University, Aarhus C 8000, Denmark

[b]Department of Materials and Environmental Chemistry, Stockholm University, Stockholm 114 18, Sweden

[c]School of Chemical Engineering, University of New South Wales, Sydney 2052, Australia

[d]Division of Surface and Corrosion Science, School of Engineering Sciences in Chemistry, Biotechnology and Health, KTH Royal Institute of Technology, Stockholm 100 44, Sweden

[e]Australian Centre for Neutron Scattering, ANSTO, Lucas Heights, New South Wales 2234, Australia

[f]National Deuteration Facility, ANSTO, Lucas Heights, New South Wales 2234, Australia

[g]Department of Engineering Design, KTH Royal Institute of Technology, Stockholm 100 44, Sweden

[h]Department of Electromechanical, Systems and Metal Engineering, Ghent University, Ghent 9052, Belgium

[i]School of Chemistry, University of New South Wales, Sydney 2052, Australia

[j]Laboratoire de Tribologie et Dynamique des Systèmes, École Central de Lyon, Lyon 69130, France

Emails for all corresponding authors:^

[*] **anja-verena.mudring@bce.au.dk**

[#] **mark@kth.se**

Contents

Experimental Details S3

Materials and methods S3

SANS of EHL/acetonitrile mixtures S6

Synthesis of racemic 2-ethylhexyl laurate-d_{40} (D-EHL) S7

References S15

Figures & Tables

Fig. S1. Small-angle neutron scattering (SANS) data of isotopic EHL mixtures presented as a log-log plot. S4

Fig. S2 Total scattering intensity from SANS fits (black right triangles); fitted coherent scattering scale (blue circles; i.e. compositional fluctuations); fitted incoherent scattering intensity from Debye's expression (light blue upward triangles) and incoherent scattering from linear interpolation of the high-Q scattering intensity of the pure H-EHL and D-EHL data, as a function of D-EHL mole fraction (blue left triangles). Lines are to guide the eye; fitting[2,3] was impossible due to the lack of literature isothermal compressibility (χ_T) data for 2-EHL. S5

Fig. S3. ^{1}H-NMR of (2-ethylhexyl) laurate-d_{40}. S8

Fig. S4. ^{2}H-NMR of (2-ethylhexyl) laurate-d_{40}. S9

Fig. S5. ^{13}C{^{1}H, ^{2}H}-NMR of (2-ethylhexyl) laurate-d_{40}. S10

Fig. S6. Expanded view of ^{13}C{^{1}H, ^{2}H}-NMR of (2-ethylhexyl) laurate-d_{40}. S11

Fig. S7. ^{13}C{^{1}H}-NMR of (2-ethylhexyl) laurate-d_{40}. S12

Fig. S8. Expanded view of ^{13}C{^{1}H}-NMR of (2-ethylhexyl) laurate-d_{40}. S13

Fig. S9. Mass spectrum of 2-EHL-d_{40}; used to calculate the overall deuteration level. S14

Table S1. SANS fitting parameters for the cylindrical model used to fit data for H-EHL dispersed in d_3-acetonitrile. S6

Experimental Details

Materials and methods

Dichloromethane was distilled prior to use. Lauric-d_{23} acid was prepared by hydrothermal deuteration. 2-Ethylhexan-1-ol-d_{17} was produced by hydrothermal deuteration of 2-ethylhexanoic-d_{15} acid, followed by reduction with lithium aluminium deuteride. All other solvents and reagents were used as received from commercial vendors. Column chromatography was performed using a Büchi Pure Chromatography System. Infrared absorption spectra were recorded on a Thermo Scientific NicoletTM iSTM10 FTIR spectrometer using neat compound, and the data are reported as wavenumbers (cm^{-1}). Nuclear magnetic resonance spectra were recorded at 300 K using a Bruker AVANCE DRX400 (400 MHz) spectrometer equipped with a 5 mm PABBO BB H/D z-gradient probe. ^{1}H chemical shifts are expressed as parts per million (ppm) with residual chloroform (δ 7.26), as reference and are reported as chemical shift (δ), relative integral – for example: 4.65 (1 residual H). ^{13}C chemical shifts are expressed as parts per million (ppm) with residual chloroform (δ 77.16) as reference and reported as chemical shift (δ); multiplicity – for example: 38.23 (CD_2). ^{2}H chemical shifts are reported as parts per million (ppm) with external referencing and are given as chemical shift (δ); multiplicity – for example: 4.63 (2 D). ^{13}C resonances attached to deuterium appear as multiplets when only the proton nucleus is decoupled (^{13}C {^{1}H}) and resolve to singlets when both proton and deuterium nuclei are decoupled (i.e., ^{13}C {^{1}H,^{2}H}). Low-resolution mass spectrometry (LRMS) was recorded using atmospheric-pressure chemical ionization (APCI) on an Advion expression Compact Mass Spectrometer. The overall percentage deuteration of the molecules was calculated by mass spectrometry using the isotope distribution analysis of the different isotopologues, including correction factors calculated for the isotopic distribution of non-hydrogen atoms.

SANS measurements were performed on the beamline BILBY, located at the ANSTO reactor source (Lucas Heights), Sydney, Australia. Samples of D-EHL and H-EHL were mixed, then transferred and sealed into 1 mm quartz Hellma© disk-shaped “banjo” cuvettes for measurement at 25°C. BILBY is a versatile SANS instrument with ToF capability; here, to achieve the most pristine background signal, the instrument was operated in monochromatic mode (λ = 4.5 Å) and in a customised distribution of rear and curtain detectors, yielding a Q-range of 0.01 – 0.72 $Å^{-1}$ in a fixed configuration

which allows for a single measurement, rather than merging data taken across multiple detector distances. The measured raw neutron scattering data (integration, masking, and subtraction of background signal) were treated using Mantid. Analysis was performed by implementing the Debye model described in Equation 1 (main body text) in Python, applying the curve fitting algorithm of SciPy for least-squares optimisation.[1]

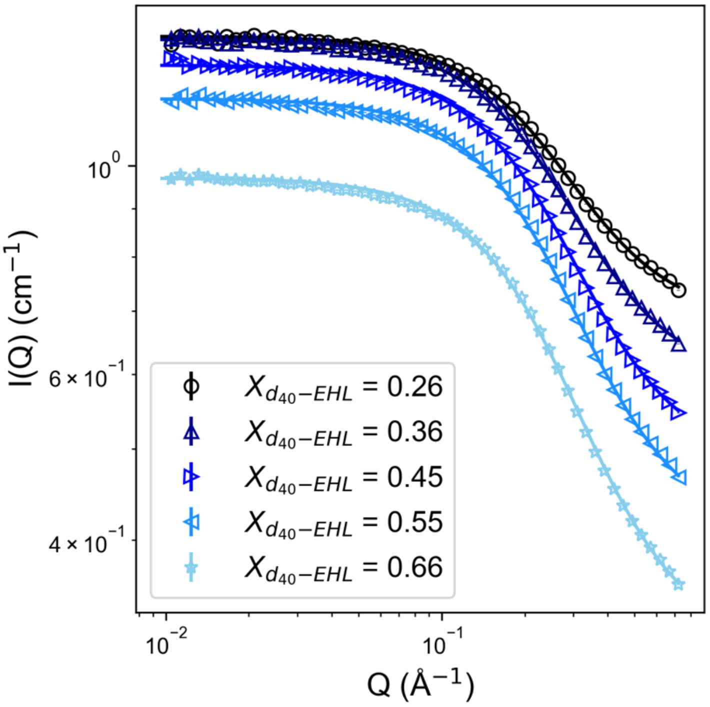

Fig. S1. Small-angle neutron scattering (SANS) data of isotopic EHL mixtures presented as a log-log plot.

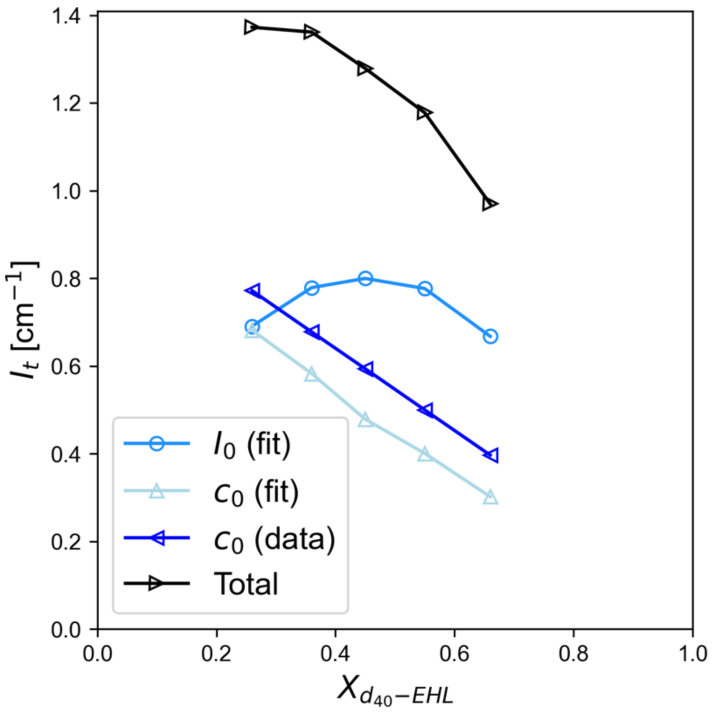

Fig. S2. Total scattering intensity from SANS fits (black right triangles); fitted coherent scattering scale (blue circles; i.e. compositional fluctuations); fitted incoherent scattering intensity from Debye's expression (light blue upward triangles) and incoherent scattering from linear interpolation of the high-Q scattering intensity of the pure H-EHL and D-EHL data, as a function of D-EHL mole fraction (blue left triangles). Lines are to guide the eye; fitting[2,3] was impossible due to the lack of literature isothermal compressibility (χ_T) data for 2-EHL.

SANS of EHL/acetonitrile mixtures

H-EHL was mixed with d_3-acetonitrile (ACN; Cambridge Isotopes, 99.5% chemical purity and 99.8 atom% D) and homogenized using a vortex mixer. Samples were transferred into quartz Hellma cells and measured using the BILBY diffractometer in an identical configuration to that used for isotopic EHL mixtures. Following corrections, a background measurement of d_3-acetonitrile was subtracted.

After trialling several models, data were found to fit very well to a form factor describing a simple cylinder, which has been described elsewhere previously.[4] The scattering length density for calculated and used for the d_3-acetonitrile solvent was 4.92×10^{-6} Å^{-2}, and for H-EHL this was -0.08×10^{-6} Å^{-2}. The fitted micelle dimensions were used to obtain the volume of the cylindrical scattering objects, from which an aggregation number was determined using the calculated molecular volume of EHL of 603.67 Å^3, i.e. $N_{agg} = \frac{\pi R^2 L}{V}$, where R is the fitted radius, L is the fitted length, and V is the molecular volume of a single EHL molecule.

Table S1. SANS fitting parameters for the cylindrical model used to fit data for H-EHL dispersed in d_3-acetonitrile.

Φ_{EHL}	Scale	c_0 (cm^{-1})	Radius (Å)	Length (Å)	Volume (Å^3)	N_{agg}
0.01	0.004	0.010	5.95±0.15	18.97±0.53	2107	3.5
0.04	0.017	0.034	5.24±0.04	23.58±0.13	2031	3.4
0.05	0.024	0.046	5.20±0.03	26.34±0.10	2240	3.7

Synthesis of racemic 2-ethylhexyl laurate-d_{40} (D-EHL)
To a solution of lauric-d_{23} acid (10.1 g, 45 mmol, 1.0 equiv.) and 2-ethylhexan-1-ol-d_{17} (7.0 g, 47 mmol, 1.1 equiv.) in CH_2Cl_2 (200 mL) was added 4-dimethylaminopyridine (0.55 g, 4.5 mmol, 0.10 equiv.). The mixture was stirred, cooling to 0 °C. A solution of dicyclohexylcarbodiimide (10.3 g, 50 mmol, 1.1 equiv.) in CH_2Cl_2 (50 mL) was added at 0 °C, with stirring, over 0.5 h. The solution was stirred at 0 °C (1 h), then allowed to warm gradually to ambient temperature, with stirring (16 h). Hexane (250 mL) was added to the stirred solution and the resulting suspension filtered through a pad of celite, washing with hexane (50 mL). The filtrate was concentrated in vacuo and the residue subjected to automated flash column chromatography (0→30% CH_2Cl_2 in hexane) to give the product (13.8 g, 87%, d. 0.971 g cm^{-3})† as a near-colourless oil; ^{1}H NMR (400 MHz, $CDCl_3$) 3.90 – 3.97 (2 residual H), 2.18 – 2.30 (2 residual H), 1.54 – 1.60 (2 residual H), 1.47 – 1.54 (1 residual H), 1.11 –1.32 (26 residual H), 0.74 – 0.88 (9 residual H); ^{2}H NMR (61 MHz, $CDCl_3$) 3.63 – 4.12 (2 D), 1.97 – 2.42 (2 D), 1.40 – 1.76 (3 D), 0.92 – 1.39 (26 D), 0.60 – 0.92 (9 D); $^{13}C\{^1H,^2H\}$ NMR (101 MHz, 400 MHz, $CDCl_3$) 174.30 (1 C), 66.00 (1 CD_2), 37.75 (1 CD), 33.77 (1 CD_2), 30.64 (1 CD_2), 29.32 (1 CD_2), 28.40 (2 CD_2), 28.27 (1 CD_2), 28.13 (1 CD_2), 28.08 (1 CD_2), 27.99 (1 CD_2), 27.70 (1 CD_2), 24.10 (1 CD_2), 22.70 (1 CD_2), 21.79 (1 CD_2), 21.49 (1 CD_2), 13.04 (1 CD_3), 12.97 (1 CD_3), 9.96 (1 CD_3); $^{13}C\{^1H\}$ NMR (101 MHz, 400 MHz, $CDCl_3$) 174.28 (1 C), 65.43 – 66.62 (1 CD_2), 37.33 – 38.36 (1 CD), 33.19 – 34.32 (1 CD_2), 30.13 – 31.37 (1 CD_2), 27.24 – 30.05 (8 CD_2), 23.56 – 24.75 (1 CD_2), 20.98 – 23.39 (3 CD_2), 12.32 – 13.75 (2 CD_3), 9.34 – 10.58 (1 CD_3); IR (neat, cm^{-1}) 2214, 2197, 2098, 1734, 1278, 1086, 1056; MS (ESI$^+$), quoted as: mass (relative intensity): 375 ($[MNa]^+$ = $[C_{20}D_{40}O_2Na]^+$, 95%), 374 (100), 373 (47), 372 (15), 371 (4).

†Mass spectrometric data, provided in Figure S7, were used to determine an overall deuteration level of 97.4±2%-d, and thereby calculate a formula weight of 351.74 g•mol^{-1}.

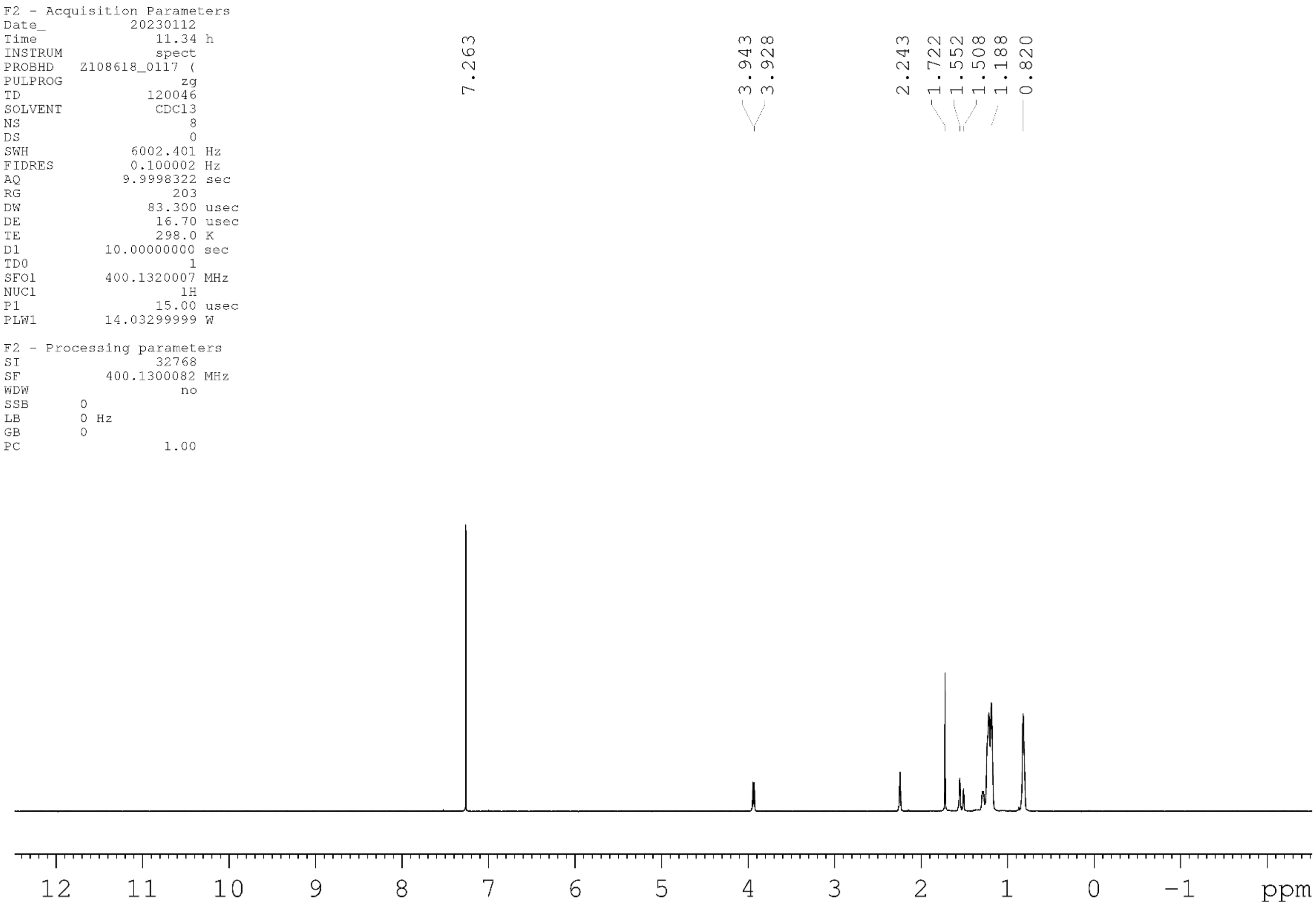

Fig. S3. ^{1}H-NMR of (2-ethylhexyl) laurate-d40.

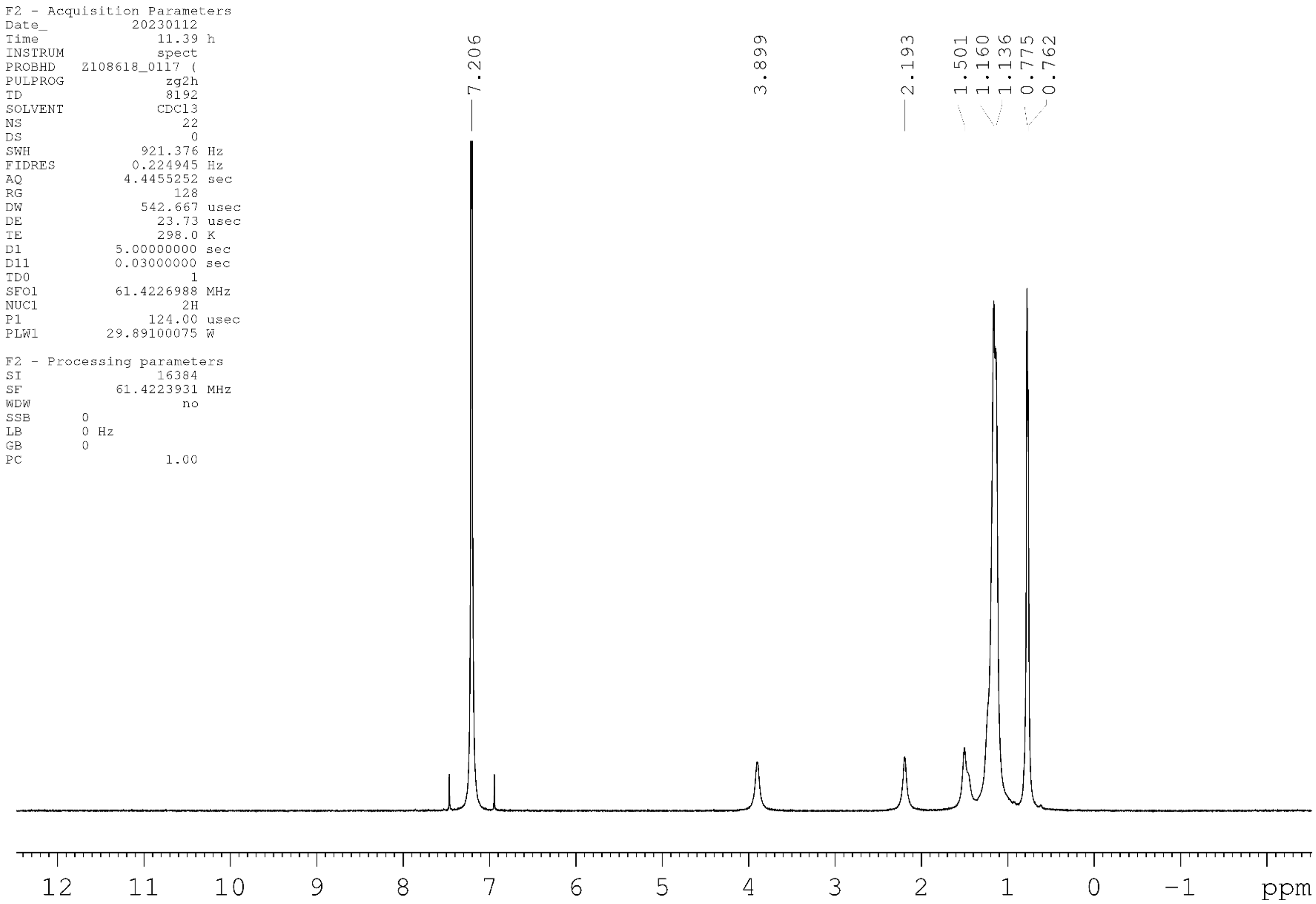

Fig. S4. ^{2}H-NMR of (2-ethylhexyl) laurate-d_{40}.

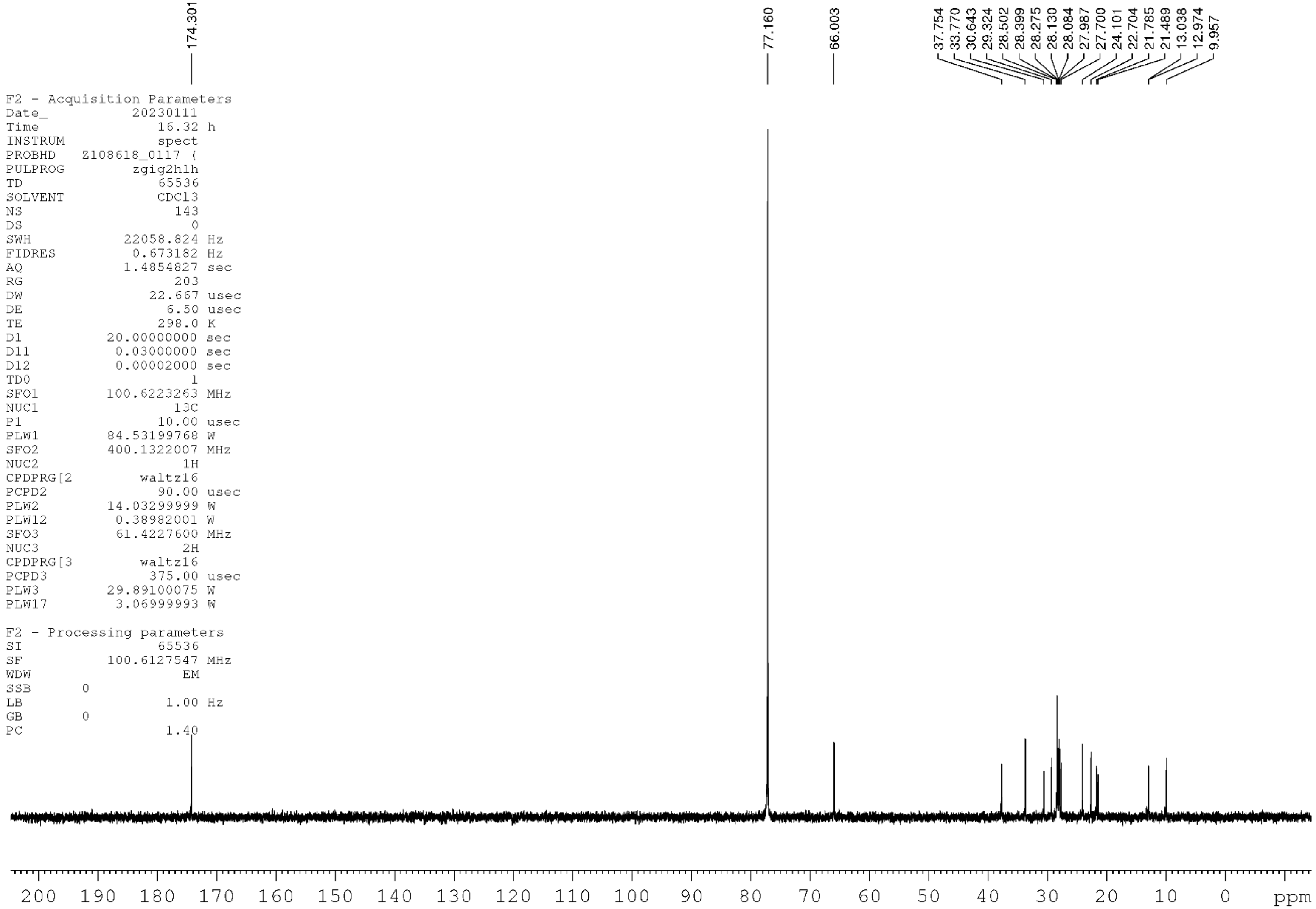

Fig. S5. $^{13}C\{^1H, ^2H\}$-NMR of (2-ethylhexyl) laurate-d_{40}.

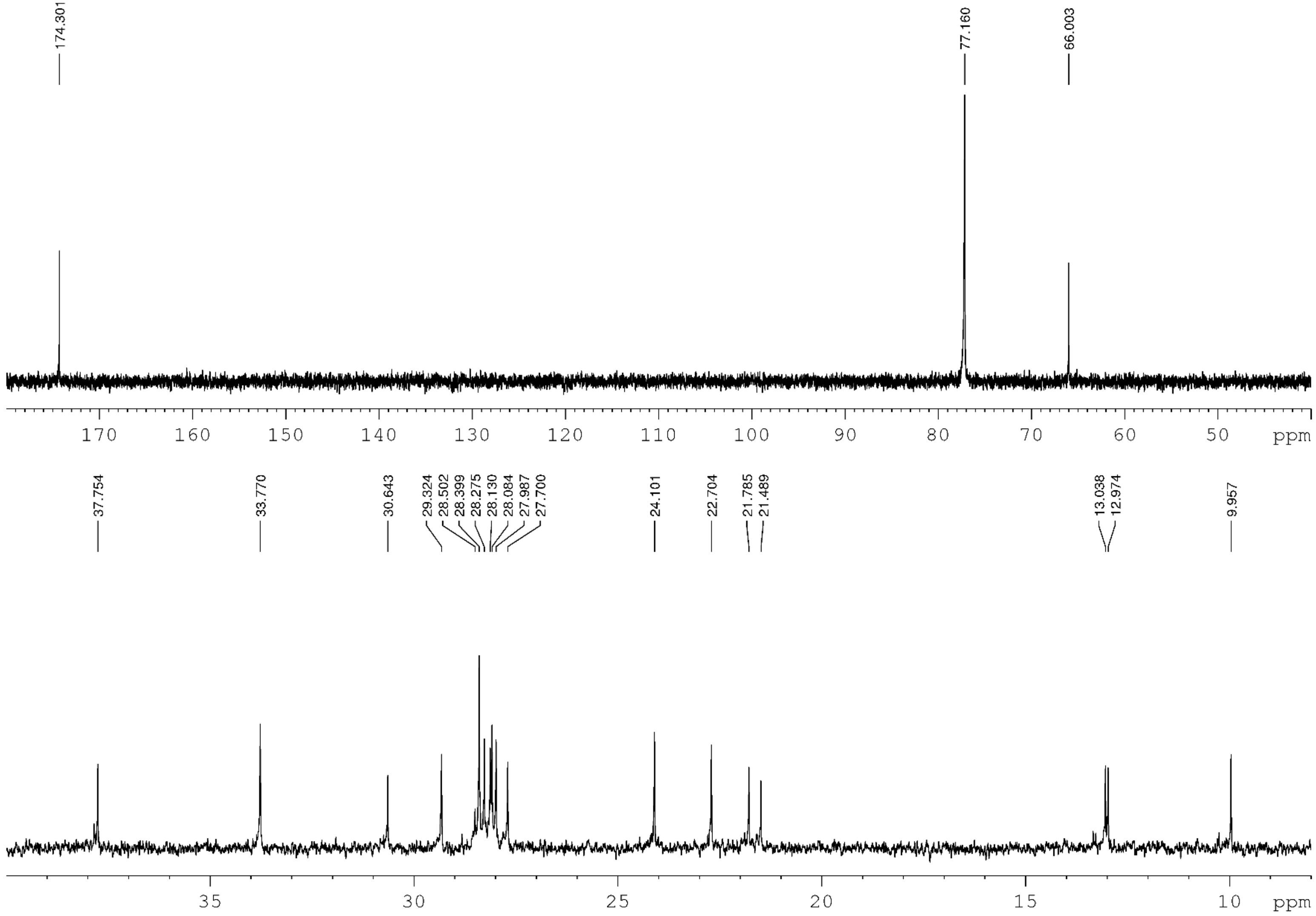

Fig. S6. Expanded view of $^{13}C\{^{1}H, ^{2}H\}$-NMR of (2-ethylhexyl) laurate-d_{40}.

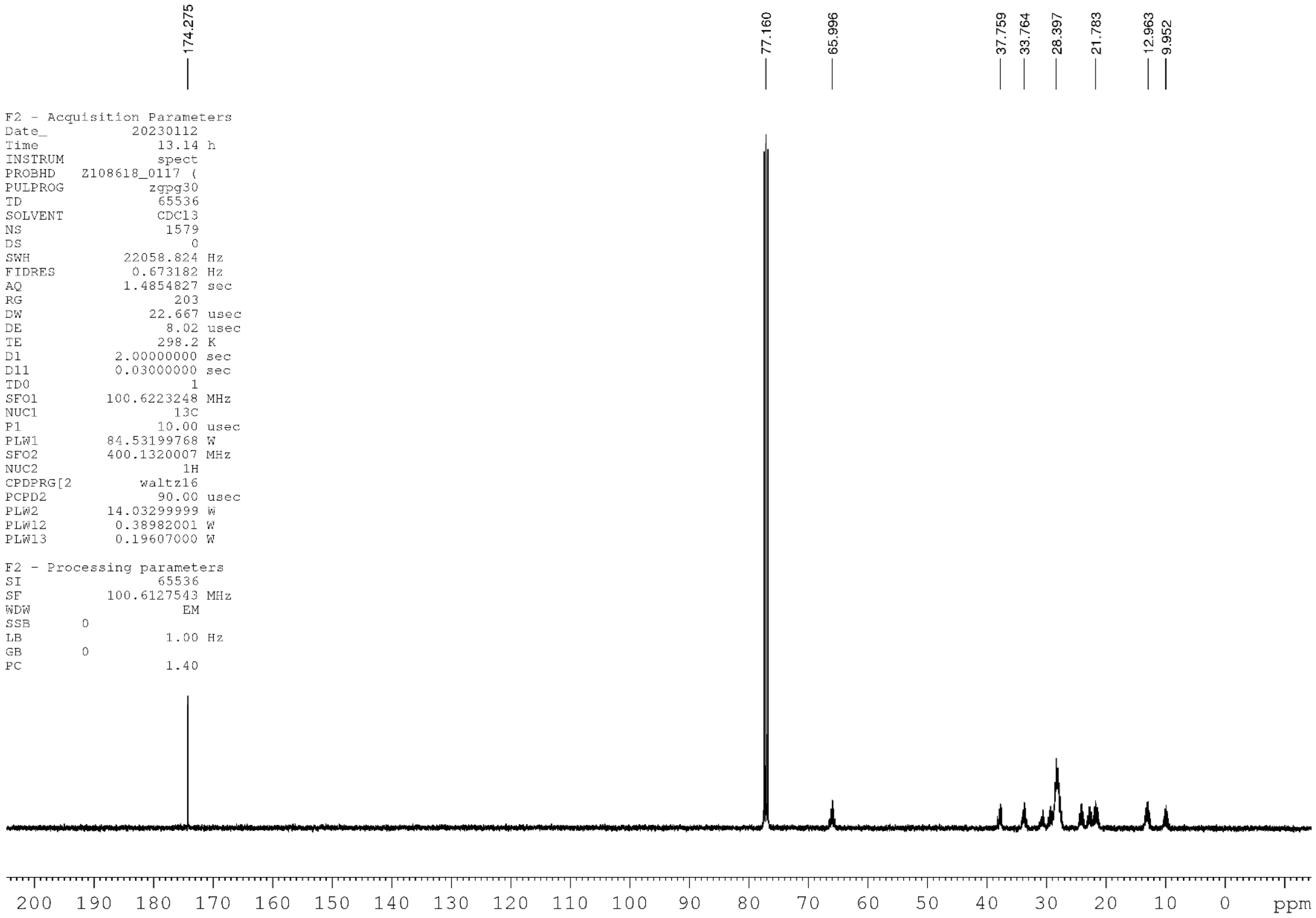

Fig. S7. $^{13}C\{^{1}H\}$-NMR of (2-ethylhexyl) laurate-d_{40}.

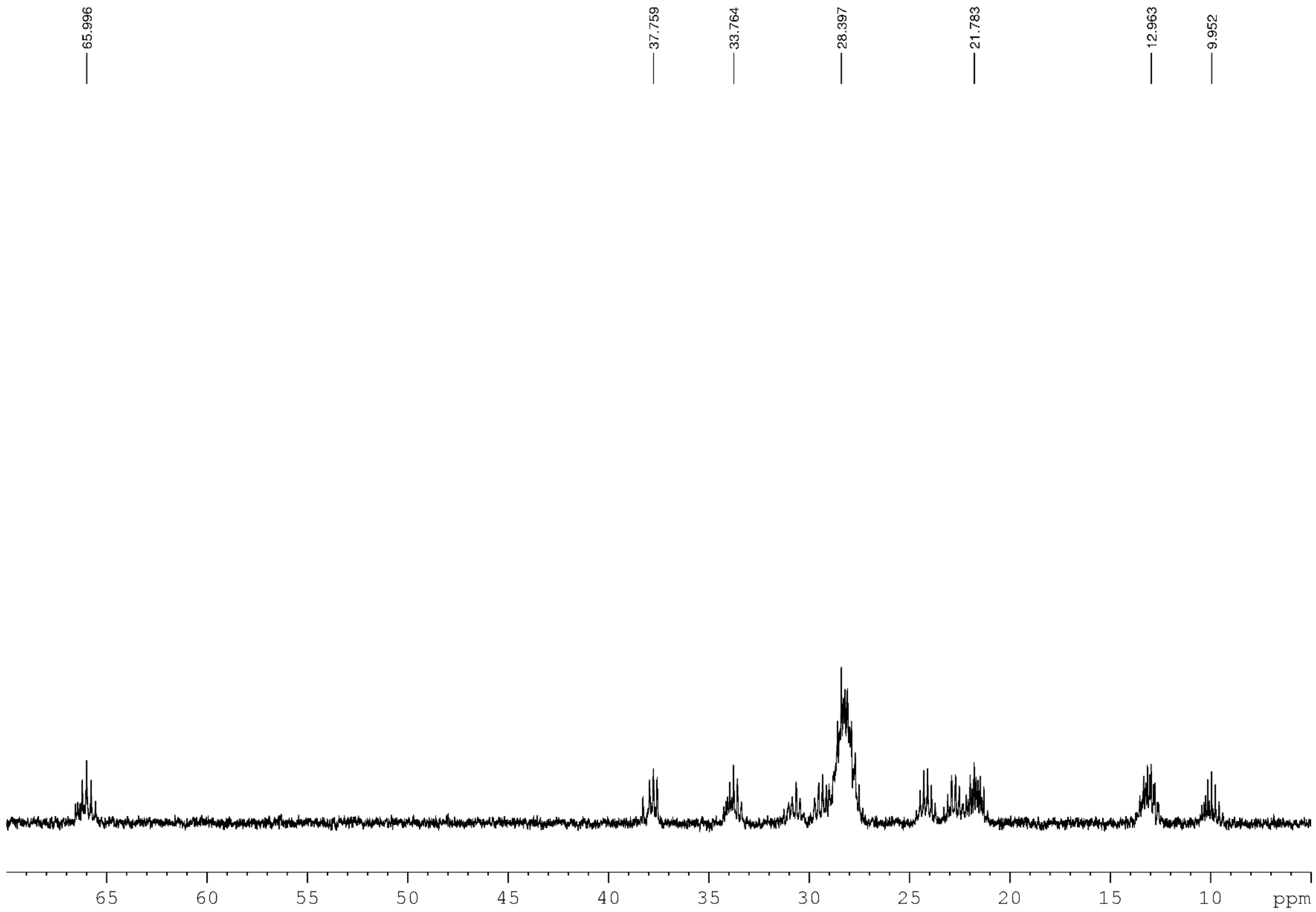

Fig. S8. Expanded view of $^{13}C\{^1H\}$-NMR of (2-ethylhexyl) laurate-d_{40}.

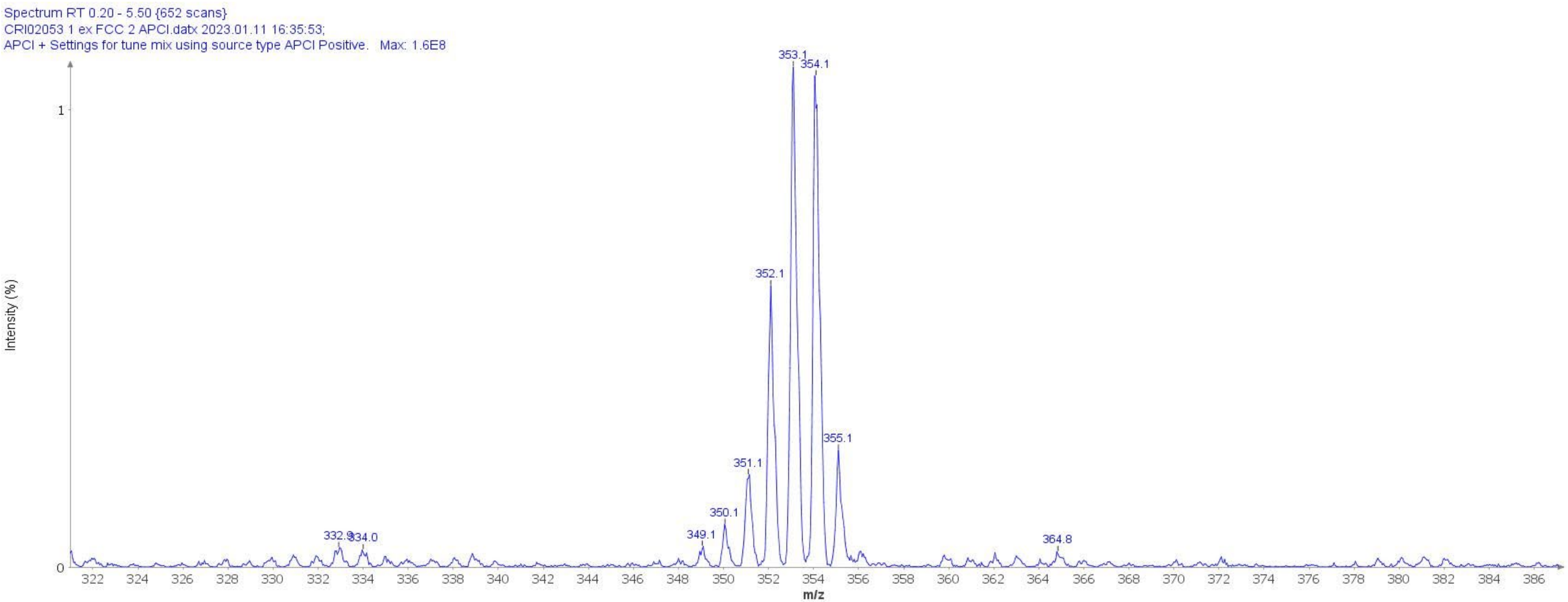

Fig. S9. Mass spectrum of 2-EHL-d_{40}; used to calculate the overall deuteration level.

References

(1) Virtanen, P.; Gommers, R.; Oliphant, T. E.; Haberland, M.; Reddy, T.; Cournapeau, D.; Burovski, E.; Peterson, P.; Weckesser, W.; Bright, J.; van der Walt, S. J.; Brett, M.; Wilson, J.; Millman, K. J.; Mayorov, N.; Nelson, A. R. J.; Jones, E.; Kern, R.; Larson, E.; Carey, C. J.; Polat, İ.; Feng, Y.; Moore, E. W.; VanderPlas, J.; Laxalde, D.; Perktold, J.; Cimrman, R.; Henriksen, I.; Quintero, E. A.; Harris, C. R.; Archibald, A. M.; Ribeiro, A. H.; Pedregosa, F.; van Mulbregt, P.; SciPy 1.0 Contributors; Vijaykumar, A.; Bardelli, A. P.; Rothberg, A.; Hilboll, A.; Kloeckner, A.; Scopatz, A.; Lee, A.; Rokem, A.; Woods, C. N.; Fulton, C.; Masson, C.; Häggström, C.; Fitzgerald, C.; Nicholson, D. A.; Hagen, D. R.; Pasechnik, D. V.; Olivetti, E.; Martin, E.; Wieser, E.; Silva, F.; Lenders, F.; Wilhelm, F.; Young, G.; Price, G. A.; Ingold, G.-L.; Allen, G. E.; Lee, G. R.; Audren, H.; Probst, I.; Dietrich, J. P.; Silterra, J.; Webber, J. T.; Slavič, J.; Nothman, J.; Buchner, J.; Kulick, J.; Schönberger, J. L.; de Miranda Cardoso, J. V.; Reimer, J.; Harrington, J.; Rodríguez, J. L. C.; Nunez-Iglesias, J.; Kuczynski, J.; Tritz, K.; Thoma, M.; Newville, M.; Kümmerer, M.; Bolingbroke, M.; Tartre, M.; Pak, M.; Smith, N. J.; Nowaczyk, N.; Shebanov, N.; Pavlyk, O.; Brodtkorb, P. A.; Lee, P.; McGibbon, R. T.; Feldbauer, R.; Lewis, S.; Tygier, S.; Sievert, S.; Vigna, S.; Peterson, S.; More, S.; Pudlik, T.; Oshima, T.; Pingel, T. J.; Robitaille, T. P.; Spura, T.; Jones, T. R.; Cera, T.; Leslie, T.; Zito, T.; Krauss, T.; Upadhyay, U.; Halchenko, Y. O.; Vázquez-Baeza, Y. SciPy 1.0: Fundamental Algorithms for Scientific Computing in Python. Nat. Methods **2020**, 17 (3), 261–272. https://doi.org/10.1038/s41592-019-0686-2.

(2) Smith, G. N.; Prevost, S. Small-Angle Neutron Scattering Measurements of Mixtures of Hydrogenous and Deuterated n-Tetradecane. J. Appl. Crystallogr. **2021**, 54, 541–547. https://doi.org/10.1107/S1600576721001138.

(3) Arleth, L.; Pedersen, J. S. Scattering Vector Dependence of the Small-Angle Scattering from Mixtures of Hydrogenated and Deuterated Organic Solvents. J. Appl. Crystallogr. **2000**, 33 (3), 650–652. https://doi.org/10.1107/S0021889899012789.

(4) Pedersen, J. S. Analysis of Small-Angle Scattering Data from Colloids and Polymer Solutions: Modeling and Least-Squares Fitting I. Adv Colloid Interface Sci **1997**, 70, 171–210. https://doi.org/10.1016/S0001-8686(97)00312-6.
